# Delayed post-traumatic left gastric artery pseudoaneurysm presenting with massive upper gastrointestinal hemorrhage: a case report

**DOI:** 10.1097/RC9.0000000000000548

**Published:** 2026-09-08

**Authors:** Harroum Meriem, Harbi Raida, Letaief Amal, Sghaier Asma, Elghali Mohamed Amine

**Affiliations:** a Department of General Surgery, Farhat Hached University Hospital, Faculty of Medicine Ibn Al Jazzar, Sousse, Tunisia; b Department of Gastroenterology and Digestive Endoscopy, Farhat Hached University Hospital, Faculty of Medicine Ibn Al Jazzar, Sousse, Tunisia

**Keywords:** aneurysm, case reports, embolization, false, gastric artery, gastrointestinal hemorrhage, operative, surgical procedures, therapeutic

## Abstract

**Introduction and importance:**

Post-traumatic visceral artery pseudoaneurysms are rare vascular entities but carry a high risk of catastrophic hemorrhage. They most commonly involve the hepatic artery, while pseudoaneurysms of the left gastric artery are exceptionally uncommon. Delayed presentation several years after trauma is particularly rare and poses significant diagnostic challenges.

**Presentation of case:**

We report the case of a 37-year-old woman who presented with massive hematemesis and hemodynamic instability. She had a history of childhood abdominal trauma following a road traffic accident requiring partial splenectomy and reported vague abdominal pain in the weeks preceding the event. Initial differential diagnoses included peptic ulcer bleeding and esophageal varices. Upper gastrointestinal endoscopy revealed a large amount of intragastric blood without an identifiable source. Contrast-enhanced CT angiography demonstrated a saccular pseudoaneurysm of the left gastric artery with tortuous vessels. During preparation for endovascular embolization, the patient developed recurrent massive hemorrhage requiring emergency laparotomy. Intraoperative findings confirmed rupture into the gastric lumen, and hemostasis was achieved by ligation of the left gastric artery. The postoperative course was uneventful, and the patient was discharged in stable condition.

**Clinical discussion:**

Visceral artery pseudoaneurysms should be considered in unexplained gastrointestinal bleeding, particularly in patients with prior abdominal trauma. CT angiography is essential for diagnosis and guides management. Endovascular embolization is preferred in stable patients, whereas active rupture requires urgent surgical intervention.

**Conclusion:**

Delayed post-traumatic pseudoaneurysms should be included in the differential diagnosis of severe gastrointestinal hemorrhage. Early recognition, prompt imaging, and timely intervention are crucial to improve outcomes.

## Introduction

Visceral artery pseudoaneurysms are rare, with a reported incidence of approximately 0.1–0.2%, although this is likely underestimated due to asymptomatic cases^[^1^]^. They most commonly involve the hepatic artery and its branches^[^2^]^, while gastric artery pseudoaneurysms account for less than 4% of cases, with left gastric artery pseudoaneurysms representing the rarest subtype. Despite their rarity, these lesions carry a high risk of rupture, with mortality rates ranging from 20% to 70% following hemorrhagic events^[^2,3^]^.

HIGHLIGHTS

- Post-traumatic visceral artery pseudoaneurysms are rare but potentially life-threatening.
- Pseudoaneurysm should be considered in the differential diagnosis of patients presenting with unexplained gastrointestinal bleeding and a history of abdominal trauma.
- The left gastric artery is an exceptionally rare site of visceral artery pseudoaneurysm formation.
- Contrast-enhanced CT angiography is essential for diagnosis and therapeutic planning.
- Endovascular embolization is safe and effective in hemodynamically stable patients, whereas open surgery remains mandatory in cases of hemodynamic instability or an active rupture.

Most cases are clinically silent and incidentally detected on cross-sectional imaging, whereas presentation with acute gastrointestinal hemorrhage is rare^[^4^]^. Vascular-related gastrointestinal bleeding is more commonly attributed to gastroduodenal artery aneurysms or pseudoaneurysms^[^5^]^, while involvement of the left gastric artery remains exceptional, with only a limited number of cases have been reported^[^6–8^]^.

The exceptionally long latency period of 27 years following abdominal trauma makes this case highly unusual. Reporting such cases is important, as diagnosis may be challenging and delayed, especially in patients with unexplained gastrointestinal bleeding. Although endovascular embolization is considered first-line treatment in stable patients, management strategies are not standardized and depend on clinical context and available resources. To date, no specific international guidelines exist for the management of visceral artery pseudoaneurysms^[^9^]^.

This case report has been reported in accordance with the SCARE checklist^[^10^]^.

## Case presentation

We report the case of a 37-year-old woman who self-presented to the emergency department of a tertiary care hospital with massive gastrointestinal hemorrhage manifested as profuse hematemesis. She had a history of blunt abdominal trauma during childhood following a road traffic accident requiring partial splenectomy, with no postoperative complications. She reported vague abdominal pain in the weeks preceding the event, without melena or prior bleeding and with no identifiable triggering factor. She had no significant medical history or relevant drug allergies, and she denied smoking, alcohol, or drug use.

On presentation, she was conscious, pale, tachycardic (120 bpm), and hypotensive (90/50 mmHg), consistent with severe hypovolemia. Laboratory tests revealed severe anemia (hemoglobin of 6.5 g/dL), elevated urea (12 mmol/L), and normal liver tests and prothrombin time.

Resuscitation included transfusion of four units of packed red blood cells and intravenous crystalloids, resulting in hemodynamic improvement and a hemoglobin increase to 9.5 g/dL.

Upper gastrointestinal endoscopy performed 6 hours after admission revealed a large amount of intragastric blood (~200 mL aspirated) without an identifiable bleeding source. Initial differential diagnosis included peptic ulcer bleeding and esophageal varices; however, the absence of an identifiable mucosal source on endoscopy suggested a vascular etiology.

Contrast-enhanced CT angiography performed within 1 hour demonstrated a saccular pseudoaneurysm of the left gastric artery (Fig. 1) with tortuous vessels along the lesser curvature of the stomach (Fig. 2).

**Figure 1.**
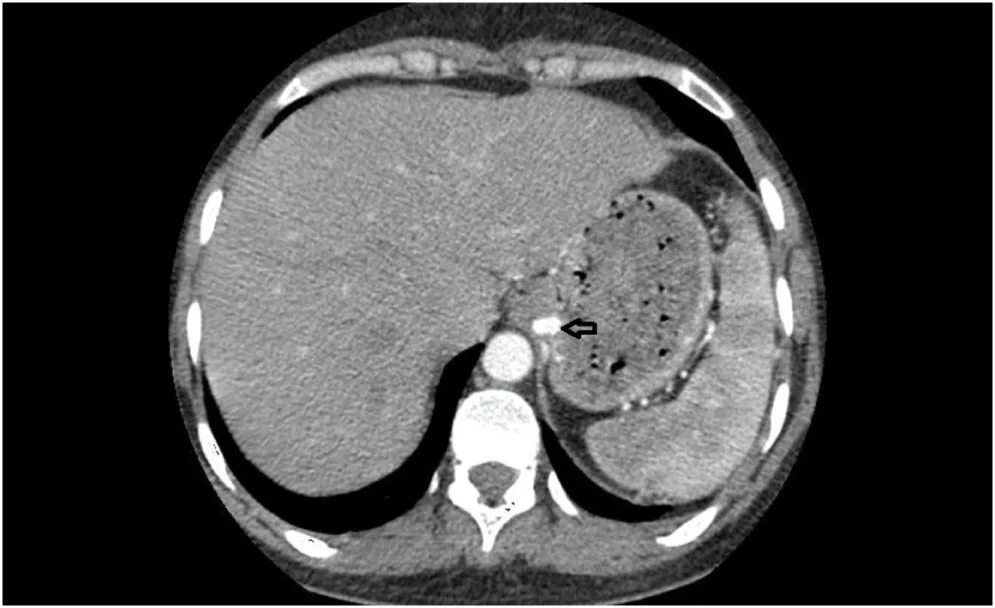
Contrast-enhanced abdominal computed tomography (CT) angiography showing a saccular pseudoaneurysm of the left gastric artery (arrow).

**Figure 2.**
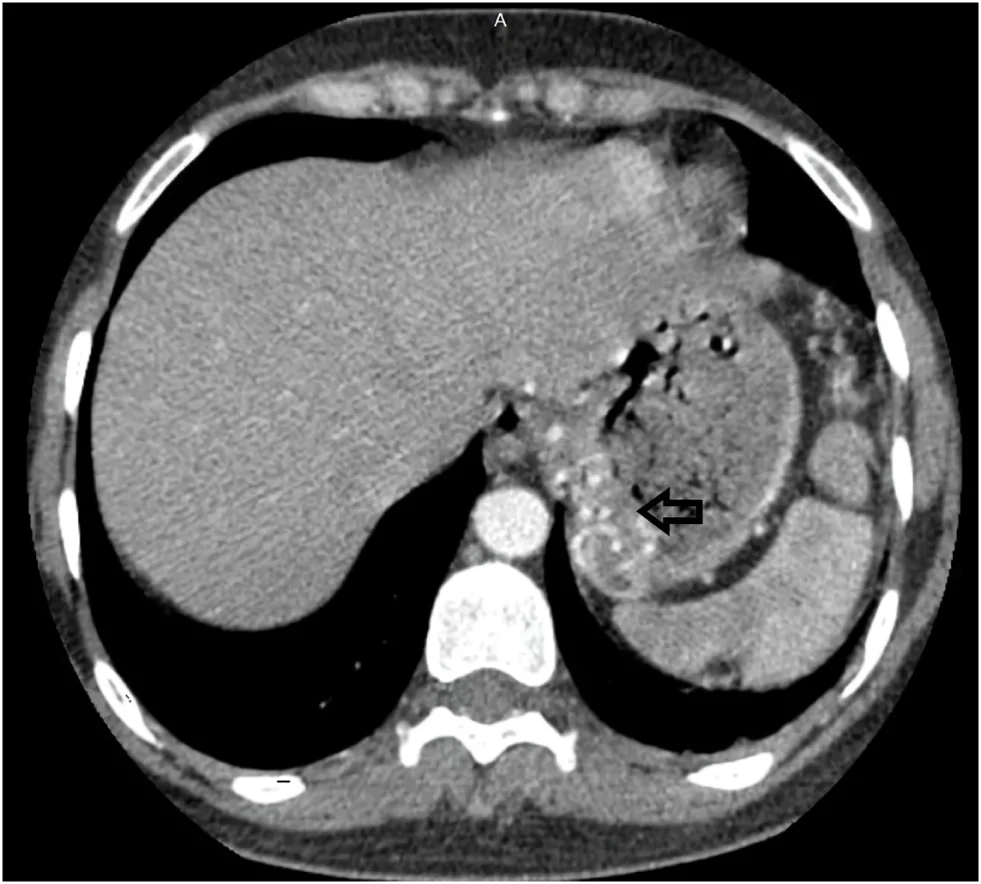
Tortuous vascular structures along the lesser curvature of the stomach (arrow).

Endovascular embolization was planned shortly after imaging; however, the patient developed recurrent massive hemorrhage approximately 2 hours later, requiring emergency surgery within the next hour.

Emergency laparotomy was performed under general anesthesia. Tranexamic acid was administered to support hemostasis. A longitudinal gastrotomy over the lesser curvature exposed active, pulsatile bleeding originating from a non-ulcerated mucosal defect (Fig. 3), consistent with rupture of the pseudoaneurysm into the gastric lumen. Initial local suturing attempts failed to achieve hemostasis, and definitive bleeding control was obtained by double ligation of the left gastric artery approximately 30 minutes after the incision. Blood loss was approximately 500 mL and was managed with blood transfusion and crystalloid infusion.

**Figure 3.**
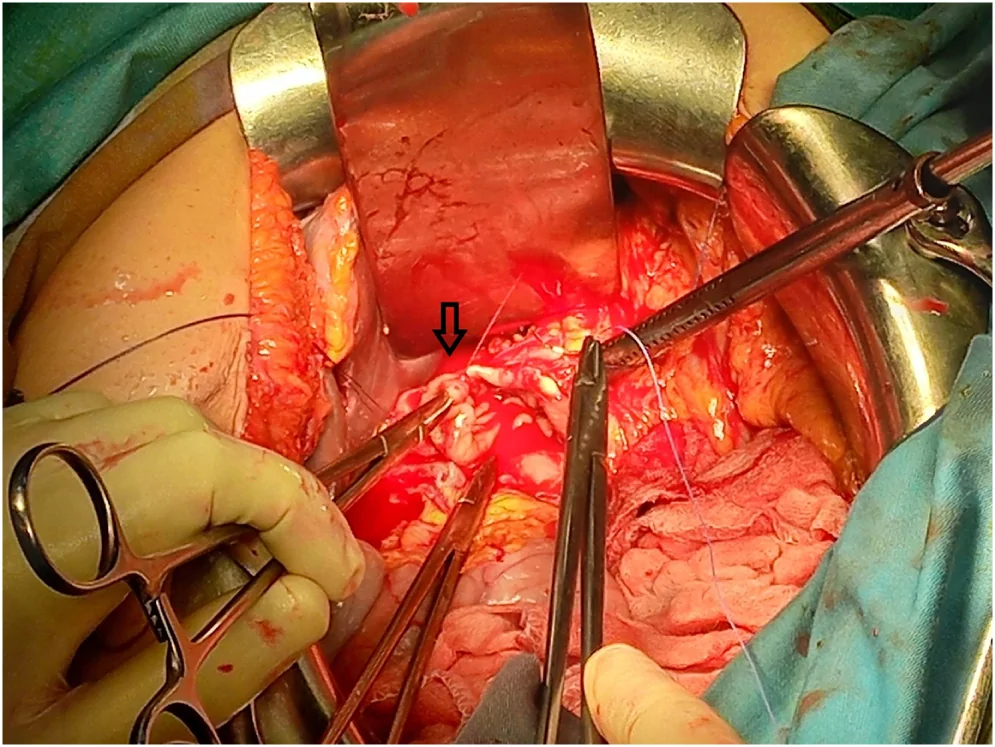
Intraoperative view showing active jet-like hemorrhage (arrow) from a ruptured left gastric artery pseudoaneurysm fistulizing into the gastric lumen.

The patient required transient low-dose norepinephrine, which was discontinued a few hours postoperatively following hemodynamic stabilization; the patient was extubated after 24 hours. Postoperative management included nasogastric decompression, intra-abdominal drainage, and a short course of antibiotics.

The postoperative course was uneventful. After 48 hours in the intensive care unit, she was transferred to the surgical ward and discharged home on postoperative day four.

Follow-up in the outpatient clinic by the surgical team at 15 days and at 1 month showed no recurrence of bleeding, with stable hemoglobin levels and no complications. Gradual return to normal activity was advised, with no need for specific rehabilitation or psychological support, and long-term follow-up was planned.

## Discussion

Gastrointestinal (GI) hemorrhage is the most frequent emergency in hepatogastroenterology. Acute upper GI bleeding accounts for approximately 80% of cases^[^11^]^, most commonly due to peptic ulcer disease or esophageal varices. Vascular lesions are less frequent but important causes of hematemesis^[^11,12^]^.

Visceral artery aneurysms and pseudoaneurysms are rare, with a prevalence of 0.1–0.2%, but are potentially life-threatening causes of gastrointestinal bleeding. Pseudoaneurysms carry a significantly higher risk of rupture than true aneurysms (76.3% vs. 3.1%)^[^10^]^, and are associated with high mortality rates of 20–70%^[^2,3^]^.

Iatrogenic causes have become increasingly common with the widespread use of percutaneous and endovascular procedures. They are most often reported after hepatobiliary and pancreatic surgeries and are related to vascular injury, enzymatic degradation, bile leakage, or intra-abdominal sepsis^[^1^]^.

Aneurysmal erosion into the gastrointestinal tract is most commonly reported in hepatic or gastroduodenal artery pseudoaneurysms^[^2,5^]^. Post-traumatic pseudoaneurysms are less frequent and usually present shortly after injury^[^13^]^, although delayed presentations reflecting progressive vascular remodeling may occur. Initial arterial injury may lead to a contained hematoma that progressively expands under hemodynamic stress into a pseudoaneurysm^[^14,15^]^. Rupture may occur spontaneously due to progressive wall weakening and can result in massive hemorrhage^[^1,6^]^.

In our case, the exceptionally long latency of 27 years suggests a slow evolution. Prior splenectomy may have contributed by altering regional hemodynamics and increasing blood flow through collateral pathways, including the left gastric artery. However, the exact etiology remains unclear, whether it is related to the initial trauma or to long-term hemodynamic changes after splenectomy.

Upper GI bleeding due to rupture of a left gastric artery pseudoaneurysm remains exceptionally rare, with only a limited number of cases reported^[^6–8^]^. The main distinctive feature of our case is the unusually long delay between childhood trauma and rupture.

CT angiography is the gold standard for diagnosis and guides management. Endoscopic treatment remains first-line for most upper GI bleeding but is often insufficient in vascular lesions such as pseudoaneurysms^[^16^]^. In such cases, management relies on endovascular embolization or surgery, depending on hemodynamic status^[^11,15^]^.

In our patient, embolization was planned; however, recurrent hemorrhage required emergency surgery.

This case highlights the importance of considering vascular etiologies in unexplained GI bleeding, particularly in patients with prior abdominal trauma. Early recognition and timely imaging are essential to improve outcomes.

### Strengths

This case describes an exceptionally rare cause of massive upper gastrointestinal hemorrhage: a delayed post-traumatic pseudoaneurysm of the left gastric artery. The long latency and unusual vascular location enhance its clinical relevance. The report emphasizes the diagnostic value of CT angiography when endoscopy is non-diagnostic and highlights effective multidisciplinary management in a life-threatening setting.

### Weaknesses and limitations

The main limitation is the single-case design, which restricts generalizability. Endovascular embolization could not be performed because of rapid hemodynamic deterioration, thereby precluding comparison with surgical treatment. Although effective in stable patients, embolization may be contraindicated in cases of active rupture or instability, where emergency surgery remains the definitive treatment.

## Conclusions

Although uncommon, post-traumatic visceral artery pseudoaneurysms should be considered in both acute and long-term follow-up of patients with prior abdominal trauma. A high index of suspicion is essential due to the risk of delayed presentation and catastrophic rupture. Endovascular embolization is preferred in hemodynamically stable patients with suitable anatomy; however, surgical intervention remains mandatory in cases of instability or active rupture.

## Data Availability

Data supporting this case report are included in the article. No additional data are available.
